# On the Decomposition of the Bile of the Ox

**Published:** 1850-03

**Authors:** 


*On the Decomposition of the Bile of the Ox.* By Dr. Buchner, jun.—M. de Gorup-Besanez, has been for some time studying, in Dr. Buchner's laboratory, the series of alterations which the bile undergoes when removed from the economy. The mucus of the bile first becomes decomposed, and supplies a ferment which determines the decomposition of the fluid itself, the products of which fermentation bear a strong analogy to those which result from the action of acids and alkalies on the bile. Thus, by the action of hydrochloric acid, the essential portion of the bile is decomposed into *choloidic acid*, which is precipitated, and into two neutral and crystallizable bodies, *glycocoll* and *taurine*, which remain dissolved with the chloride of soda, &c. Under the operation of alkalies, in the place of *choloidic acid*, there is formed the *cholic* of Demarcay (the *chololic* of Strecker); and if a strong alkali be employed, as caustic potass, in place of glycocoll and taurine, we have the products of their decomposition—ammonia, &c. So, too, after the first period of the putrefactive fermentation of bile, which is terminated in from four to six weeks, beautiful crystals of taurine may be separated; and a resinous precipitate produced by the action of acids, after separating the fatty matters from the residue, and redissolving it in water, is found to be of the same composition as *choloidic acid;* while, if the bile has been fermented at a low temperature, crystals of *chololic acid* are produced—these two acids, indeed, only differing from each other by one equiv. of H. O. Another product due to the formation and decomposition of *glycocoll*, is ammonia, the greatest portion of which escapes during evaporation. Thus, the first phasis of the fermentation, which may be called the *bilic* fermentation, furnishes—

1. Ammonia, } Products containing all the azote, and all the sulphur
2. Taurine, } of the fermented bile.
3. The resinoid choloidic or chololic acid, combined with soda and ammonia, and precipitable by a stronger acid.

A further fermentation, which may be called the *tauric*, is effected at the expense of the taurine. Prisms of *sulphate of soda* are soon observed, and as this salt does not exist in the fresh bile, it can only result from the decomposition of the taurine, the only sulphurous body formed during the *bilic* fermentation. The quantity of taurine gradually diminishes *pari passu* with the increase of that of this salt, which eventually entirely displaces it. The same is observed in bile which has been purified by concentrated alchohol, in which the sulphates, owing to their insolubility could not have existed. The *sulphate* is not produced directly from the decomposition of the taurine, but by the successive oxidation of the hyposulphite, or sulphite which is so. In putrefied bile, too, we may always detect acetic acid, and other volatile acids of analogous composition, especially valerianic.—*Brit. & For. Med. Chir. Rev. from Jour. de Pharmacie.*

